# Determinants of Contraceptives Use amongst Youth: An Exploratory Study with Family Planning Service Providers in Karachi Pakistan

**DOI:** 10.5539/gjhs.v5n3p1

**Published:** 2013-01-05

**Authors:** Noureen Aleem Nishtar, Neelofar Sami, Sabina Alim, Nousheen Pradhan, Farid-Ul-Hasnain

**Affiliations:** 1 Community Health Sciences Department, Aga Khan University Karachi, Pakistan; 2 Willows Foundation Pakistan, Pakistan; 3 Rawal Medical College, Rawalpindi, Pakistan; 4 Community Health Sciences Department, Aga Khan University Karachi, Pakistan

**Keywords:** contraceptives, youth, Pakistan, intra uterine contraceptive device, oral contraceptive pills, contraceptive injections, family planning service providers

## Abstract

**Introduction:**

In Pakistan, Contraceptive Prevalence Rate (CPR) among married female youth is 17.4% and even lower in rural and slum areas leading to rapid population growth on one hand and poor health consequences on the other. The study was conducted to explore family planning service providers’ perceptions regarding use of different contraceptive methods and to identify factors that are influencing their use amongst currently married youth aged 18-24 years in slum areas of Karachi.

**Method:**

Qualitative exploratory study design was adopted and a total of ten in-depth interviews were conducted with family planning service providers of the area. For content analysis coding of transcribed interviews was done and then categories were made and furthermore themes were derived.

**Results:**

Our findings revealed that family planning service providers perceived that there is low use of contraceptive methods amongst youth of study area and low usage could be due to side effects; myths and misconceptions; lack of proper knowledge about different contraceptives; unmet needs of contraceptives; socio-cultural and religious factors about different contraceptive methods and family planning service providers own biases against or for use of contraceptive methods amongst youth in the study area. However better education of youth and family planning service providers’ improved knowledge about counseling and use of contraceptive methods was perceived to be associated with improved use of family planning methods amongst youth of the study area.

**Conclusion:**

Exaggerated side effects and socio-cultural factors could be important influences leading to low use of family planning methods amongst youth of Karachi. Some policy initiatives are the training of lady health Workers, lady health visitors, physicians and staff of the pharmacies for counseling youth in the correct use of family planning methods.

## 1. Background

Being one of the poorest and sixth populous countries of the world (World fact book, 2011), Pakistan has had very slow progress on the Contraceptive Prevalence Rate (CPR) which is 29.6% with the use of modern contraceptive methods at only 21% (PDHS 2006-07). CPR among married female youth in the country is even lower than national average at 17.4 percent and even lower at 13.2 % in rural areas (UNFPA, 2011). Pakistan along with other developing countries like India, Nigeria, and Congo contributed to 50% of all maternal deaths across the globe (Hogan et al., 2011; Alam, 2011). Of those, an estimated 52 maternal deaths occur daily in rural Pakistan, based on the urban-rural divide (Azmat et al., 2012). Many studies have indicated that family planning could be instrumental in maintaining better quality of health amongst females and could be helpful in combating maternal and neonatal mortalities and morbidities (Kols and Sherman 1998, Erim, Resch, & Goldie, 2012; Tsui, McDonald-Mosley, Burke, & Yeakey, 2009). Moreover youth comprised of large chunk of the population of Pakistan with the foremost concern being low use of contraceptives which is further adding to the population growth (Kols & Sherman, 1998; PRB, 2012). Pakistan’s low CPR has become a grave concern, because one of the major complications associated with low use of contraceptives is none other than maternal deaths due to unwanted pregnancies (Hogan et al., 2011). In this worsening scenario, adolescent pregnancies are rising across the globe (Kennedy, Gray, Azzopardi, & Creati, 2009). And adolescents are five time more likely to die of pregnancy and maternal conditions than adult women (Patton et al., 2009; WHO, 2006). Teenage pregnancies are an important indicator of reproductive health and Pakistan Demographic Health Survey has reported that 23% of women start child bearing by the age of 19 years in Pakistan and fertility is maximum in 20 to 24 years age group (PDHS 2006-07). These pregnancies among teenage youth could have been prevented by effective and consistent use of contraceptives and the resulting risk of early parenthood and unintended pregnancies being averted but the key aspect is promoting contraceptive use among youth.

To date voluminous studies have explored the determinants towards the use of different contraceptives methods among married couples across the globe (Agha & Edmeades, 2006; Grossman, Ellertson, Abuabara, Blanchard, & Rivas, 2006; Jones, 2001). For instance, in Pakistani context, husband and the mother in law have been reported to be the major family planning decision making authority (Grossman et al., 2006; Jones, 2001). Review of literature has broadly categorized three major obstacles in Pakistan towards the use of contraceptive methods: perception of women that contraceptive use would conflict with her husband’s attitude towards family planning, social and cultural acceptability as perceived by women and knowledge about the contraceptive methods (Pasha, 2001; Mahmood & Ringheim, 1996). Furthermore, a recent study on perceptions regarding various contraceptive methods in Pakistan revealed that vasectomy was perceived against men’s pride, restricted females’ mobility, fear of side effects, socio-cultural influences, religious and financial limitations have been considered as the major barriers towards the adoption of family planning methods (Ali, Rayis, Mamoun, & Adam, 1996). However limited attention has been paid towards family planning service providers’ perspectives on determinants of multiple contraceptive methods especially amongst youth. Moreover family planning providers constitute an important source of information, education and communication (IEC) about various contraceptive methods to clients. Thus it’s significant to understand provider’s perspective on determinants of different contraceptive methods amongst youth. Therefore, the aim of this study was to explore the perceptions of family planning service providers about determinants of various contraceptive methods amongst youth residing in slum areas of Karachi, Pakistan.

## 2. Methodology

### 2.1 Study Design, Setting and Period

The study was conducted in Korangi town (UC 2 and 3), Karachi, Pakistan from July to September, 2010. Exploratory qualitative study design was adopted using in-depth interviews with family planning service providers. In order to capture as many aspects and views of family planning service providers, the participants were chosen purposively (Khan et al 1991).

### 2.2 Study Participants

The study participants included: Family Planning Service Providers from the 2 Union Councils of Korangi town. They included 2 physicians, 4 Lady Health Visitors, 2 Lady Health Workers and 2 medical store drug providers. Lady Health Workers working in the area facilitated the identification of family planning service providers. Inclusion criteria being family planning service providers of UC 2 and UC 3 of Korangi town, Karachi, Pakistan and those who gave consent for IDIs.

### 2.3 Data Collection

A topic guide was used to guide the discussions (Dahlgren, Emmelin, & Winkvist, 2007). The entire data collection process was pilot-tested in an area similar in socio-demographics to the study area, so that any unforeseen flaws in the protocol could be addressed. All the interviews were conducted by the principal investigator.

**Table 1:** Characteristics of in-depth-interviews with family planning service providers

Interviews	Interviewees	No.	Venues
Interview 1 & 4	Medical Doctors	2	Clinic and NGO office
Interview 2, 3, 7 & 8	Lady Health Workers	4	Health Houses
Interview 4 & 9	Lady Health Visitors	2	First level Care Facility
Interview 5 & 10	Medical Store Drug Provider	2	Pharmacy Stores

### 2.4 Analysis

Content analysis was the strategy used for analyzing the transcribed interviews. We considered a meaning unit as words, sentences or paragraphs containing aspects related to each other through their content and context (Graneheim & Lundman, 2004). Therefore for analysis purpose, meaning units were identified and condensed meaning units were derived, coding done and furthermore categories were made and themes were derived as shown in table 2. Creating categories is the core feature of qualitative content analysis. A category is a group of content that shares a commonality (Klaus 1980).

**Table 2:** Examples of Determinants of Family Planning methods use among youth as perceived by family Planning service providers

Theme	Categories	Codes	Meaning Units
Barriers to family planning methods use	Side effects	Menstrual irregularities	-Another major reason is that OCPs and injections methods cause menstrual irregularities which are not acceptable for women.
		Infections and vaginal discharge	-Regarding IUCDs, she told us that most women have pre-conceived ideas that IUCDs cause infections & vaginal discharge
		Spreads the words about side effects	-Regarding the side effects of the various methods of family planning she said that if one women experience some of them, she spread the word all around her.
	Myths and misconceptions	Travels to heart and brain	- Popular misconception with respect to IUCDS is that it moves up to the heart and brain
		Impotent	- Men believe they will become impotent because of using family planning methods.
	Religious	Family planning is wrong	-People believe that Family planning not allowed in our religion and is wrong.
		Burial time prayers not accepted	- If a woman undergoes tubal ligation, people believe that her (Namaz-e-janaza) buri al time prayers should not be offered and accepted.
	Family Planning providers own biases	Big family	-Tubal ligation is suggested to those who already have five or six children.
		Natural family planning methods preferred	- Natural family planning methods are best and even if I suggest, it is maximum condoms.
	Poor knowledge about contraceptive methods use	Non-compliant	- Women are not compliant to the dose of OCPs and sometimes don’t take OCPs for days and then take 2 or 3 pills together.
	Socio-Cultural	Patriarchal society	- Women in our part of the world do not want to trouble their husband. In her view, such women would rather sacrifice their own health.
		(Phutkari), Alum used as contraceptive	-Powdered “phutkri”, tied in a cloth & kept in the vagina during intercourse prevents conception, she had heard from a traditional birth attendant/“Dai”

Supporters of family planning method use	Better education of youth	Better education	- Better education associated with better use of contraceptives
		Awareness	- Believed that people have misconception mainly due to the lack of awareness & education
	Better knowledge of Family planning service providers	Lactating mother	-If the female is a lactating mother then she does not give her oral contraceptive pills (OCPs) for at- least 6 months. Instead, she recommends either condoms or injectable contraception.

### 2.5 Ethical Approval

Study was approved by Ethical Review Committee (ERC) of Aga Khan University (AKU) Karachi, Pakistan. The hard copies of the interviews and all audiotapes were kept in lock and key. Verbal and written consent was sought from all study participants.

### 2.6 Trustworthiness

Transcription was done on the day of the interview to ensure that there is minimum time lapse so that the data on tapes is transcribed to the paper with the fresh memory. All of the authors were competent in the local language and the cultural meaning of the content (NN, NS, FH, NP & SA). Recordings and notes were also consulted for any verification at the end of the interview with the interviewees. Interviews were transcribed into Urdu and then translated to English by the Principal Investigator. Credibility was maintained by selection of context, informants and well-structured in- depth Interviews. Conformability was achieved through separate coding by the first and third author, whereby similarities and dissimilarities were discussed. Consensus on codes and categories was thereafter reached. During the analytical process, the first and third author thoroughly discussed the structure of codes, categories and themes. All authors read, discussed and agreed on the final categorization and themes. Two of the authors (NN and SA) agreed in the way the codes and categories were labeled and categorized, which in later stages were verified by the other two authors (FH & NS). Transferability was achieved through purposive selection of informants with different background characteristics such as profession (pharmacists, medical doctors, lady health workers and lady health visitors) educational level and experiences. Dependability was enhanced by conducting the IDIs over 2 months, to ensure that the phenomena under study did not change. All IDIs were conducted in Urdu, moderated by local researchers well versed with the context, transcribed into English by the principal investigator and analyzed within a week. Verification of transcriptions was done by the first author who listened to the audio-tapes twice. The quotations given in the study are intended to facilitate the reader’s evaluation of the creditability of results (JQM 2006).

## 3. Results

Results were obtained by content analysis keeping in mind objectives of the study. Two underlying themes emerged during the analysis: barriers of family planning methods use and facilitators of family planning methods use (Table 2). For this paper, results are described under codes, categories and themes. Ten in-depth interviews were conducted with the Family Planning service providers of the area. Their experiences were varying from 4-25 years.

On Content analysis, there appeared to be multiple determinants of use of contraceptive methods as shown in Table 2.

## Barriers of family planning methods use as shown in table 2 are:

### 3.1 Side Effects

Exaggerated side effects of different contraceptive methods were considered as one of the main reasons for discontinuation of Family Planning methods.

Family Planning service providers shared that Intra Uterine Contraceptive Device (IUCDs) has most of the side effects.

“Many women complain that IUCDs make their husbands uncomfortable during intercourse because of the thread that protrudes. Therefore, IUCDs are not well liked by the general population.” (….interview 9)

“Most women taking OCPs complain of dizziness & nausea. Even if she convinces them to take the pills they, automatically, get turned off because of the side effects.” (….interview 3)

Likewise most of the family planning service providers were of the view that youth of the area think that vasectomy is causing physical weakness in males and tubal ligation is the causing weight gain in females as a side effect.

### 3.2 Myths and Misconceptions about Different Contraceptive Methods

Family Planning service providers of the area shared that myths and misconceptions about different contraceptive methods are also potential factors for not using contraceptive methods amongst youth in the study area.

“In general, men do not realize or care for the problems that their wives are going through. They do not agree to undergo vasectomy as they are afraid it will make them impotent” …. (interview 2)

“Many of the women shun IUCDs because they believe that IUCDs can cause infections, destroy the uterus somehow & that is can travel up from the uterus into the abdomen.” …. (interview 7)

“Usually they don’t have misconceptions about injections. If they have a big family, they frequently use injections” …. (interview 4)

“After using family planning method, the ovary of females will become dry and then they will not be able to have children …. (interview 10)

Majority of family planning service providers shared that youth of the area perceive that condoms cause inflammation, infections and ulceration in both males and females. Most of the Family Planning service providers said that none of the males in the area want to opt for vasectomy as they believe that vasectomy causes physical weakness and impotency.

### 3.3 Lack of Proper Knowledge about Use of Different Contraceptives

This also emerged as one of the probable determinants for non-use of Family Planning methods amongst youth of the area as some of the Family Planning service providers shared what youth of the area perceived

“Pills are difficult to remember & these women become very upset often missing a dose & do not understand what to do next.” … (interview 6)

### 3.4 Socio-Cultural Factors

All the FP service providers were of the view that education is one of the most important factors in improving CPR. Moreover some of the FP service providers shared that certain ethnicities as Bengali and Sindhi believe that big family size is associated with more respect in the society.

Most of the family planning service providers shared that Pakistan is a patriarchal society as shared in the quote by an interviewee.

“Role of mother in law and husbands is pivotal in decision making for the use of contraceptives.” … (Interviewee 8)

### 3.5 Religious Factors

Family planning providers also shared that some people of the area believe that if someone prevents pregnancy, Allah makes them ill in some other way for example blood pressure and diabetes and there is a common belief that Family Planning is forbidden in our religion.

One of a Family Planning service provider shared that

“Youth of the area thinks that their prayers will not be accepted if they use Family Planning methods” … (Interviewee 5)

### 3.6 Family Planning Service Providers Own Biases towards Use of Family Planning Methods

Some of the family planning service providers have their own biases towards the use of contraceptive methods as stated by one of the interviewee.

“I don’t recommend contraceptive methods like IUCD, injections and pills to youth females and maximum I council them for using condoms or natural family planning methods” … (Interviewee 1)

### 3.7 Facilitators of Family Planning Methods Use

Better knowledge of Family planning service providers and improved education of youth are facilitating the use of family planning methods as discussed below.

#### 3.7.1 Better Knowledge of Family Planning Service Providers

Some of the family planning service providers themselves identified that knowledge of the family planning service providers about counseling youth for use of contraceptive methods is very important in this aspect and facilitate the use of these methods amongst youth of the area.

“Injectables are not liked by women of the area; tend to cause excessive bleeding, due to hormonal imbalance. Therefore, does not prefer injectables to anemic women.”… (Interviewee 8)

“Misconception is rampant among the females who come to her is that IUCD moves up to the stomach. I, myself believe that mostly what actually happens is that the IUCD is expelled during menstruation and these women do not realize it.”… (Interviewee 1)

#### 3.7.2 Better Education of Youth

All the interviewees unanimously agreed that mostly, if the client seeking family planning services is better educated at-least (uptill grade 10), they will understand the importance of family planning methods use and will be much more compliant to the use of contraceptive methods.

## 4. Discussion

This paper has tried to add to existing evidence on factors that support or constrain the use of family planning methods and two broad themes were identified as barriers to and supporters of the use of family planning methods amongst youth of study area. We have identified many demand side barriers to the use of family planning methods use as mentioned in the study where the author has shared that demand side barriers to health services access are abundant in low- and middle-income countries and there is a lack of evidence on ways to reduce them (Ensor & Cooper 2004). In our study, socio-cultural factors specifically patriarchal society were considered as substantial barriers against family planning methods use as perceived by the family planning service providers. This is validated by a study conducted in Maya-Quiche, where community leaders, religious leaders and husbands exert significant impact on family planning decisions and usually oppose the use of contraceptives (Ward, Bertrand, & Puac, 1992). Moreover family planning service providers’ own biases were also identified as a barrier to family planning methods, therefore training exercises in clarifying providers’ values, which can help providers to identify their own biases about barriers to contraceptives is needed. This is especially important because clients may depend on providers for information and advice about newer methods (Mantell et al., 2003). In Morocco IUDs were considered underutilized by some program managers, and despite considerable efforts to promote IUDs, including extensive training, physicians remain resilient to them (Shelton 2001). In a study on the use of condoms, training of family planning service providers was identified to reduce providers’ negative perceptions of the condoms and to strengthen the importance of individualized counseling personalized client’s needs (Mantell et al., 2003).

In family planning service providers’ perceptions, illiteracy was also identified as one of the constraining factor, whereas better education of youth were identified as a supporting factor towards the use of family planning methods use. It is thought that better educational completion operates through multiple factors to influence family planning service use, encompassing female decision-making power and increasing awareness of health services (Obermeyer, 2004). Women with no education were most likely to report psychosocial barriers to the use of family planning services. A woman’s involvement in education may also increase her exposure to the health system and improved use of services (Stephenson & Hennik, 2004). Exploration of family planning providers’ perspective yielded a meaningful insight as the information explicated from them were valuable because the data collected from their own experiences and perspectives and also based on their observations, which can provide a new lens in re-shaping the service delivery of contraceptives with emphasis on overcoming various determinants of contraceptive use among youth. Findings of a study highlights that providers’ perspectives on contraceptives use among youth emphasized a lack of awareness towards important contraceptive issues, as well as a lack of true understanding of contraception which was also validated by a study conducted in United States (Brown, Burdette, & Rodriguez, 2008).

This study addresses the reproductive health issues of youth in a community setting and in depth interviews responses explicated from Family Planning service providers were valuable and based on their observations & experiences, which is one of the strength of this study. Moreover findings documented by this study would assist in planning and undertaking policy initiatives to streamline recommendations for improving the use of different contraceptive methods amongst youth in slums of Karachi. It was a not a large population study due to paucity of funds and time due to which it has certain shortcomings, the important of which are: few interviews with the respondents were not conducted in depth due to increase patient load at their offices; extreme hot weather in the month of data collection and some of respondents unwillingness to give more time for interview. One weakness of the study might be that the data were initially collected in Urdu, translated to English and analyzed in the English version. However, a multidisciplinary team with experienced qualitative researchers took part at all stages of the research process.

In the light of findings of the study and suggestions by the interviewees during in-depth interviews, some of the recommendations to address the low use of contraceptives are that there is a need to disseminate correct information on the use of contraceptive methods. It will help in removing related misperceptions about Family Planning methods through easy to understand booklets in local languages. The initiative should also include other opinion leaders in the society such as prayer leaders, school teachers, local political and social workers. It would help change the societal mindset regarding many aspects such as baseless myths. It will also help to rectify religious and cultural misperceptions. Some urgently needed policy initiatives to improve the use of contraceptives are: lady Health Workers, lady Health Visitors, physicians and staff of the pharmacies are needed to be trained for proper counseling for correct use of family planning method. In conclusion, contraceptives use amongst youth is low in the study area; in addition lack of appropriate knowledge about contraceptives contribute for low CPR among the youth and side effects; some religious and socio-cultural beliefs about different contraceptive methods could be potential determinants contributing to low use of contraceptives.
